# Anatomy of the Campi Flegrei caldera using Enhanced Seismic Tomography Models

**DOI:** 10.1038/s41598-018-34456-x

**Published:** 2018-11-02

**Authors:** Marco Calò, Anna Tramelli

**Affiliations:** 1 Instituto de Geofísica, Universidad Nacional Autónoma de México, Cd. Universitaria, Circuito Exterior s/n, 14260, Mexico City, Mexico; 2 Istituto Nazionale di Geofisica e Vulcanologia, sezione di Napoli, Osservatorio Vesuviano, Via Diocleziano 328, 80124, Napoli, Italy

## Abstract

Campi Flegrei caldera (Southern Italy) is a densely inhabited area and suffered several unrest episodes in the last centuries. The dynamic of the caldera is highly debated because of conflicting interpretations. Here we present a detailed reconstruction of the Campi Flegrei structure obtained using the microseismicity recorded during the 1984 unrest. Enhanced Seismic Tomography models obtained with these data allow us describing seismic velocities, attenuation, and scattering patterns. Results show: (1) a plumbing system with a diameter of 1 km located between 2.3 km and 4 km depth (2) a 0.5 km thick caprock located at 2 km depth interpreted as the main structure regulating the fluid interchange between deep and shallow sectors of the caldera, (3) the shape and volume of a shallow reservoir beneath the city of Pozzuoli; this reservoir played a key role during the 1982–1984 unrest, (4) several small reservoirs beneath the main craters of the caldera. All these features fit into the debated question on magmatic or hydrothermal mechanism driving the caldera deformation resulting of crucial importance to allow a better assessment of the hazard.

## Introduction

Campi Flegrei caldera (CF, Italy) is one of the known supervolcanoes, which historically reached a magnitude 7 of the Volcanic Explosivity Index [VEI^1^].

CF is affected by a peculiar phenomenon called bradyseism, i.e. strong vertical ground deformations occurring periodically with cycles that aboard a wide range, from millimetres to meters along the centuries^2^. The last two bradyseismic crises recorded at CF occurred in 1969–72 and 1982–84^2^. The deflation period that followed the last crisis was interrupted in 2006 when the ground started rising again, and nowadays reached an overall lift of almost 45 cm^3^. Furthermore, recent geochemical studies suggest the injection of magmatic fluids into the hydrothermal system located beneath the Pozzuoli city^4,5^. These observations, led the Italian Civil Defence to increase the alert level from green to yellow in 2012.

Simplifying, four possible mechanisms have been proposed to explain the peculiar behaviour of CF: (i) the presence of a shallow magmatic chamber that pushes the lid producing periodic variation of the soil level^6^, (ii) a thermic expansion of the geothermal aquifer due to the periodic increase of heat flux coming from a near magmatic chamber or deep fluids^7^, (iii) a combination of both phenomena^8^, (iv) seismicity and caldera unrest are mainly related to decarbonation reactions and presence of supercritical fluids^9^. As a consequence, the causes of the actual dynamic of CF are still under study and the mechanisms producing the bradyseismic crises are still under debate.

At present, the ingoing unrest is characterized by a slower uplift (~45 cm in 10 years) and a lower number of earthquakes compared to the bradyseismic crisis of 1982–84^3^. Microseismicity is also involving a different focal volume. However, both unrests seem to be triggered by the uprising of fluids along pre-existing faults^10^ producing a critical variation of the fumaroles composition^4^. Otherwise, the current deformation has the same shape and location as the one of 1982–84. Therefore to understand the features responsible for the 1982–84 unrests is of crucial importance to figure out the actual situation of the CF.

The last CF eruption occurred in 1538 and was preceded by ground uplift and an increase of the seismicity rate lasted some tens of years^11^. While this eruptive event is highly documented for that period, the behaviour preceding an eruption inside the caldera is not well known.

During the 1982–84 crisis, the knowledge of this historical eruption together with the high vertical deformation recorded in the central part of the city of Pozzuoli (~1.8 m^2^), and the high number of low magnitude earthquakes recorded (more than 16,000 in 2 years), led the authorities to evacuate about 40,000 people^12^.

Since then, the attention of the scientific community and authorities increased dramatically because a new eruptive episode nowadays would lead to evacuate about 500,000 people. In addition, also without an eruption, the increase of the seismicity rate would anyway have dramatic consequences in such a populated area.

Different geophysical and geological models have been produced to understand the structure of the CF and sometimes the interpretations resulted in conflicting opinions. The studies performed after the 1984 showed that the central part of the caldera is aseismic and characterized by low P-wave velocity (Vp) and high Vp/Vs ratio in the first 3 km of depth^13^. The shallow structure of the caldera was also investigated by means of boreholes, gravity and magnetic surveys^14,15^. The strong temperature gradient measured in the wells, the low Vp and high Vp/Vs, the high P-wave attenuation, and the low gravity anomaly^16^ led to hypothesize the presence of highly water-saturated fractured rocks^17,18^. No magmatic bodies greater than 1 km^3^ were evidenced in the first 4–5 km of depth^17,18^. At ~4 km, a low Vp/Vs anomaly was interpreted by^9^ as the top of gas-enriched formations under supercritical conditions. Otherwise the joint inversion of gravimetric and deformation data^15^ suggested the presence of a penny-shaped magma intrusion at about 3 km of depth fed by a deeper magma chamber. Recent studies based on seismic attenuation models^19,20^ speculated on the presence of an aseismic hot volume at a depth of almost 4 km, which was interpreted as an intrusion occurred during the bradyseismic crisis. Their data support the model of either a caprock or a cooled intrusion acting as a barrier for fluids coming from a deeper magmatic source located below the city of Pozzuoli. The caprock hypothesis is also supported by^21^ who performed rock physics analysis on samples collected in several wells drilled during the ‘80 on the caldera rim. In particular^21^, evidenced an unusual mineralization triggered by rock-fluid interactions, which increases the mechanical properties of the caprock located at 0.7–2 km of depth. This structure together with decarbonation reactions may explain the caldera unrests without the needing of deeper magmatic bodies in the system.

Geochemical studies^4,22^ suggest the existence of a connection between the shallow hydrothermal system feeding the fumaroles and deeper regions providing large amounts of magmatic gases. Studies based on the combination of geochemical and geophysical data^4,20^ speculate that the deformation pattern of the recent unrests is due to the overlapping of short-term pulses caused by injection of magmatic fluids into the shallow hydrothermal system and a long-term process of rock heating^4^, thus attributing a key role to the presence of shallow sources of magmatic fluids (shallower than 4 km). The fluid migration countered by a caprock, or a cooled intrusion, is the cause for the deformation and seismicity recorded in 1982–84.

Geophysical models proposed at present^10,19^ and reference therein show that a correlation between the seismicity and pressure variation in the hydrothermal system exists. However the debate on the mechanism feeding the shallow hydrothermal reservoir and its implication on the surface deformation is still open^4,7,8^. Some authors^21^ invoked the hydrothermal decarbonation reactions occurring in the caprock above the seismogenic volumes. Other authors suggested a magma injection involvement, especially for the 1982–84 uplift^6^. Nevertheless, studies based on geochemical markers in the fumaroles exclude any shallow arrival of new magma for the actual unrest^3^, in contrast to what suggested by^4^, and references therein.

Most geophysical studies agree in describing a circular fractured caldera rim characterized by high P-velocity, high density, and high scattering^17,18,23^. However, the models proposed until now still do not find an agreement on the contribution of shallow magma bodies during the 1982–84 unrest^3,4,20,21,23^. Furthermore, existing seismic tomographies did not reach the sufficient resolution to corroborate the existence of small magmatic bodies^24^. Additionally, though the presence of a reinforced caprock was observed in the core samples^21^, its lateral extension is still poorly constrained. All those uncertainties on the structure of the CF affect the models proposed for explaining the bradyseismic phenomenon. Consequently, it is also difficult to make a fair estimation of the seismic and volcanic hazard.

Here we show Enhanced Seismic Tomography (EST) models of P waves velocity (Vp), P-to-S waves ratio (Vp/Vs), attenuation (Qp and Qs) and scattering (Sc) built using the data recorded during the largest bradyseismic crisis occurred in the 80’s. We applied a procedure that improves the resolution and strongly reduces the biases that typically affect the tomographic studies. Our method allows to image geological structures with linear dimension of 0.5–1.0 km. These results allow us to discriminate between the magmatic or hydrothermal mechanism that hides under the hood of the CF caldera.

Finally, the joint analysis of the different models (velocity, attenuation and scattering) calculated using the same reference grid, enables us to finely compare them allowing speculations on the rheological characteristics of the heterogeneous bodies observed.

## Results

In this work we used the local seismicity occurred between January and April 1984 in the CF area. Although a spatial migration of the seismicity was observed in that time lapse^20^, the models obtained in this work can be considered representative of the structure and, if it is the case, affected only marginally by the temporal evolution of the caldera occurred at that time.

**Figure 1:**
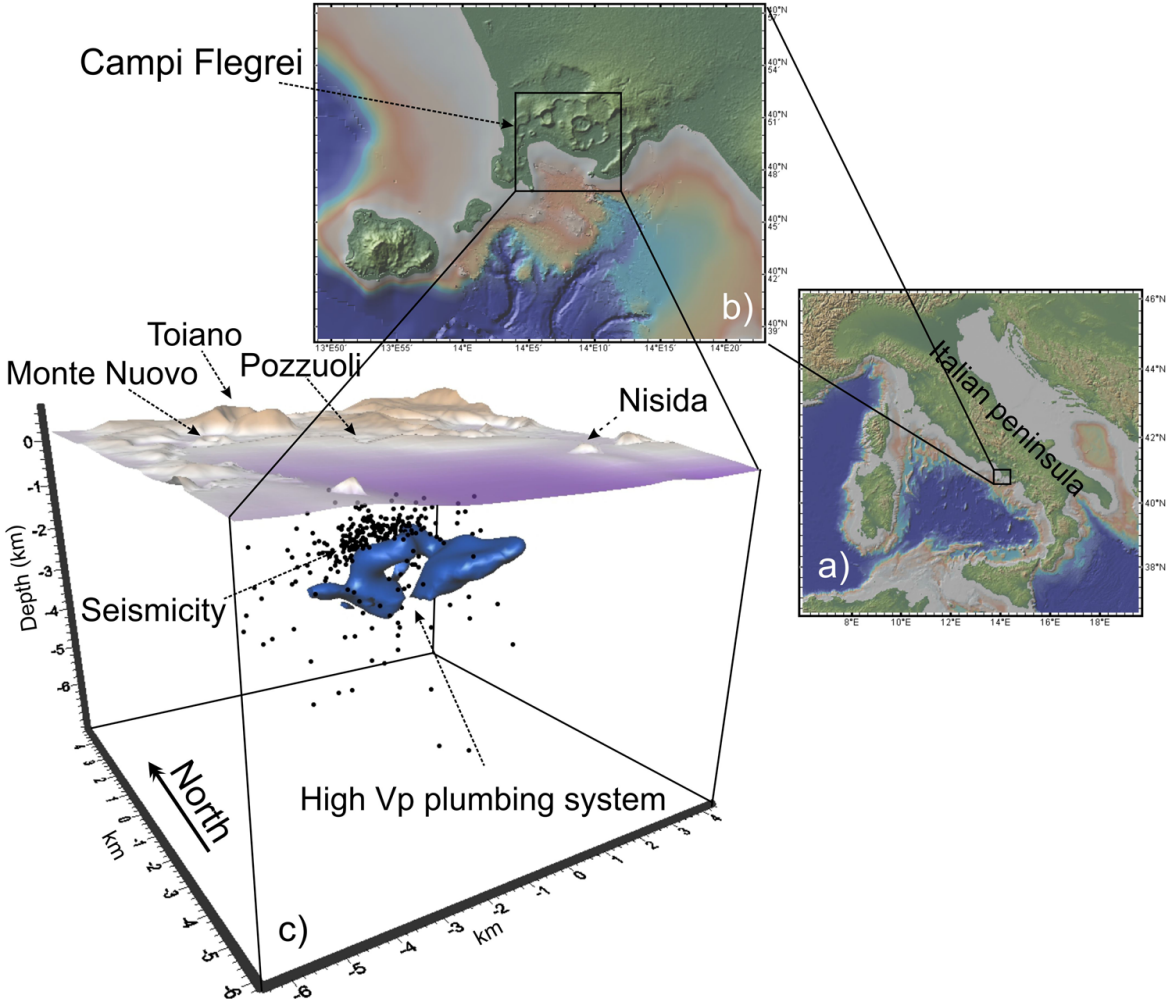
Maps with the location of the Campi Flegrei (**a**,**b**) and tridimensional view of the seismicity and of the high Vp body marked by values greater than 4.4 km/s (**c**). Maps were obtained using the open-access digital elevation model GeoMapApp^58^ (http://www.geomapapp.org/). All the 3D models were built with the software Voxler, (http://www.goldensoftware.com/products/voxler).

**Figure 2:**
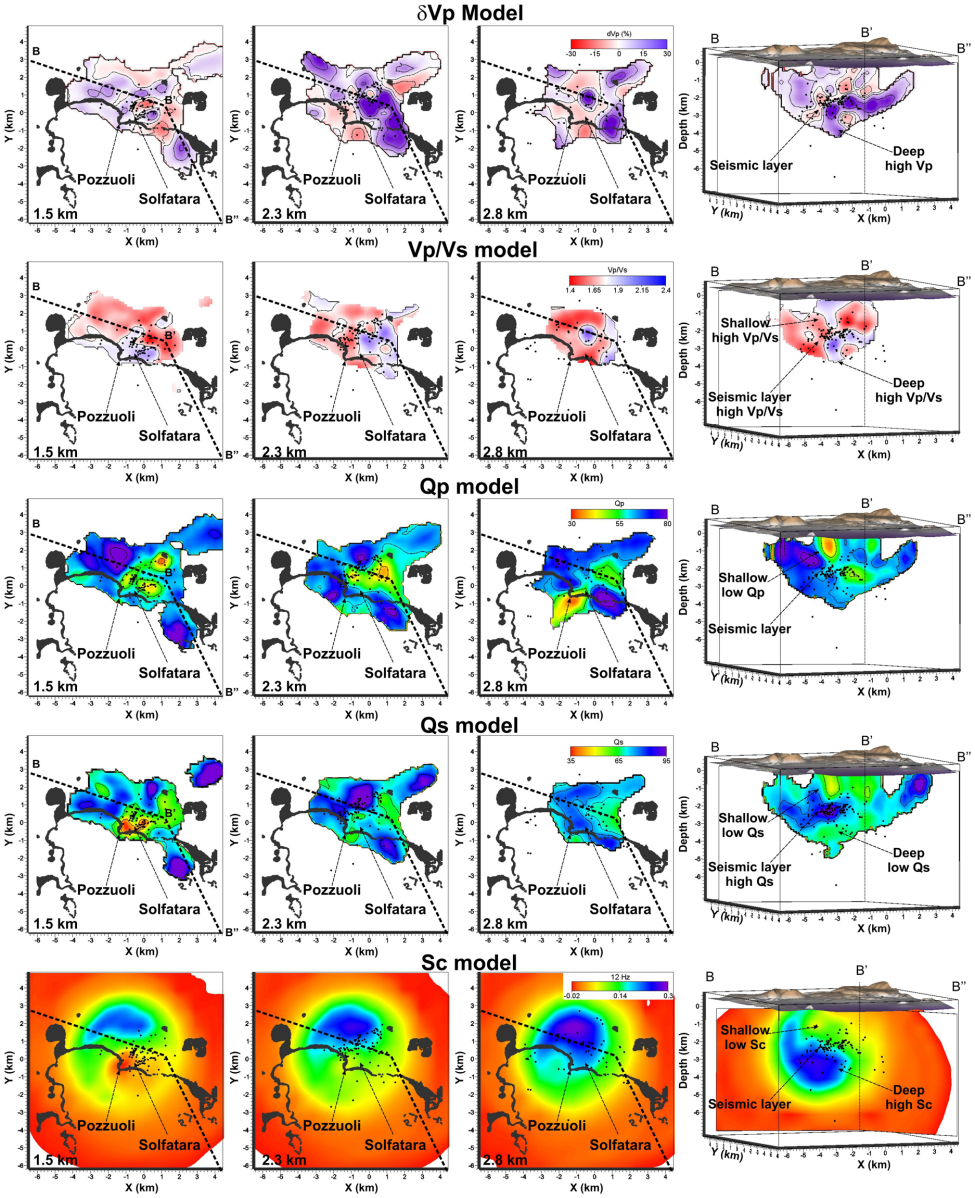
Horizontal sections of the δVp, Vp/Vs, Qp, Qs, and Sc models at 1.5 km, 2.3 km, and 2.8 km of depth. Bended cross sections of the models B-B’-B”, show de geometry of the plumbing system and its relation with the seismic layer. Black dots represent the relocated earthquakes projected onto the slices within ± 0.5 km.

**Figure 3:**
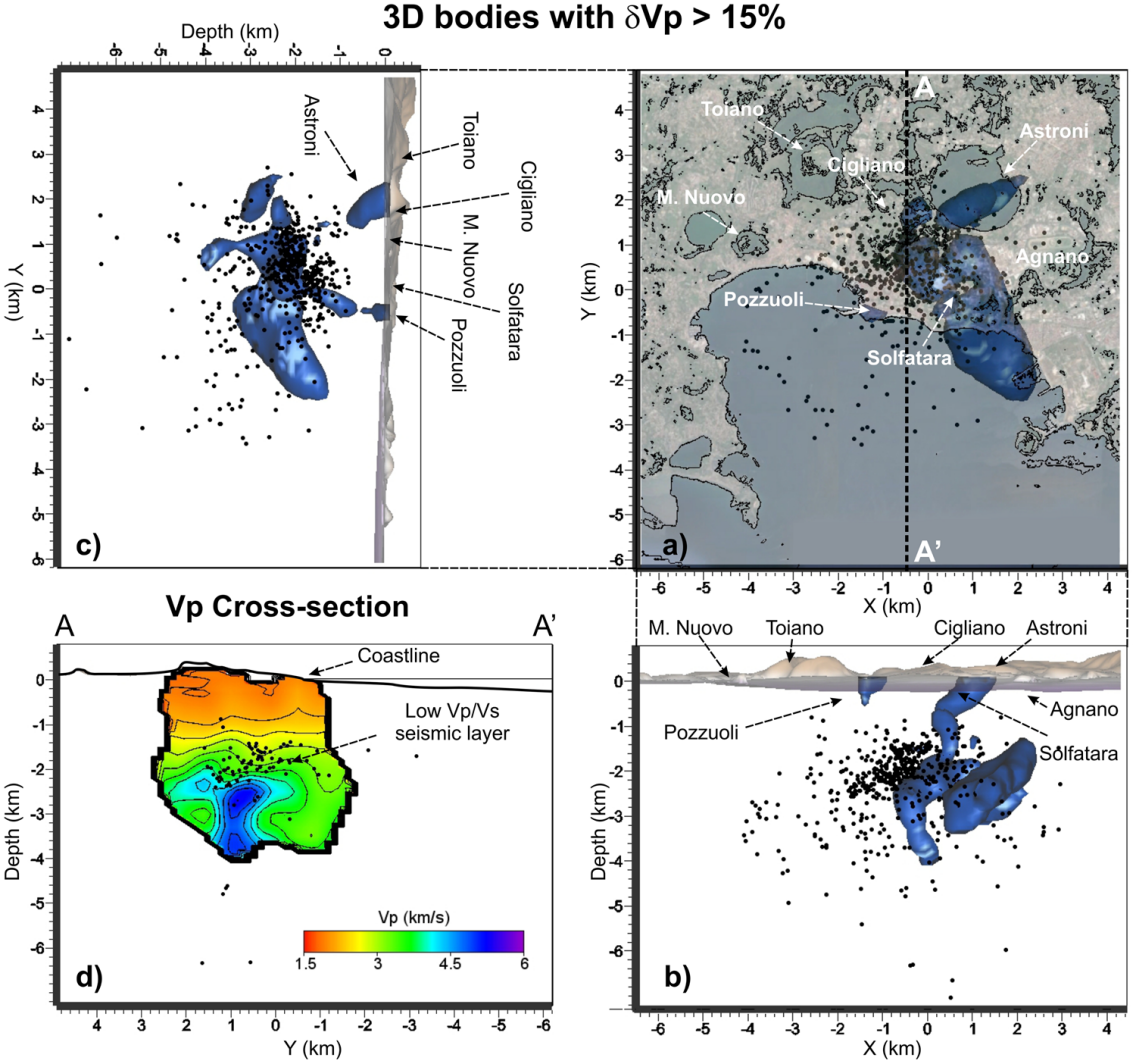
(**a**) Horizontal and (**b**,**c**) vertical projections of the seismicity and of the high Vp anomalous bodies with values greater than 15% of the initial 1D model. (**d**) Vertical cross-section of the Vp model along the profile A-A’ that displays the high Vp intrusion stopping at the seismic layer. Black dots represent the relocated earthquakes projected onto the vertical slice within ± 0.5 km.

**Figure 4:**
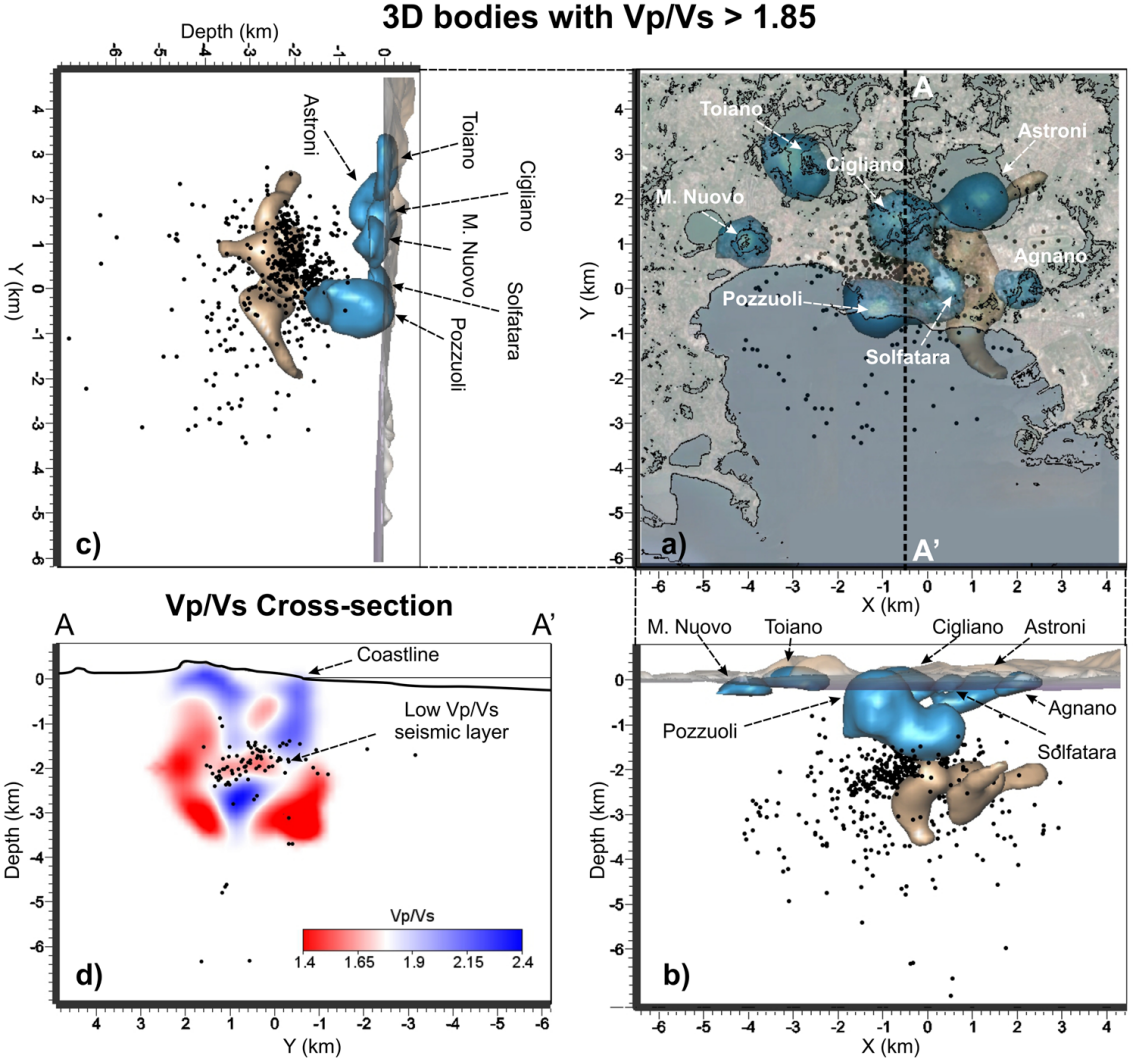
(**a**) Horizontal and (**b**,**c**) vertical projections of the seismicity and of the high Vp/Vs bodies with values greater than 1.85. Different colours are used to separate the shallow structures from the deeper ones. (**d**) Vertical cross-section of the Vp/Vs model along the profile A-A’ that evidences the low Vp/Vs seismic layer, the plumbing system and the shallow patterns. Black dots represent the relocated earthquakes projected onto the vertical slice within ± 0.5 km.

In the EST models, seismicity concentrates mainly in a thin (~0.5 km) layer located at 1.7–2.2 km of depth dividing the CF caldera in two main domains. (Fig. 1, section B-B’-B” of Fig. 2, Figs 3 and 4).

Horizontal and vertical sections of the Vp/Vs model (Figs 2 and 4) show that the medium is characterized by low values (<1.65) where high Vp/Vs bodies (>1.85) stand out. At shallow depths (<0.5 km) the high Vp/Vs bodies are mostly located beneath the major volcanic cones (Fig. 4a,b). The largest one, with a volume of ~5 km^3^, involves the inland part of the Pozzuoli region extending down to about 2 km of depth (Fig. 4b,c). Figure 2 shows a slice of this high Vp/Vs anomaly at 1.5 km depth where the location of this anomaly beneath Pozzuoli and the Solfatara crater is well noticed. At this depth, at least two fault systems striking NNE-SSW and ENE-WSW are quite well imaged (Fig. 2 at 1.5 km and numbers 1 and 2 in Fig. S1.1 of Supplementary information). This clustered seismic pattern is no longer observed at greater depths (Fig. S1.1 of Supplementary information) suggesting that these faults affect only the shallowest part the caldera.

**Figure 5:**
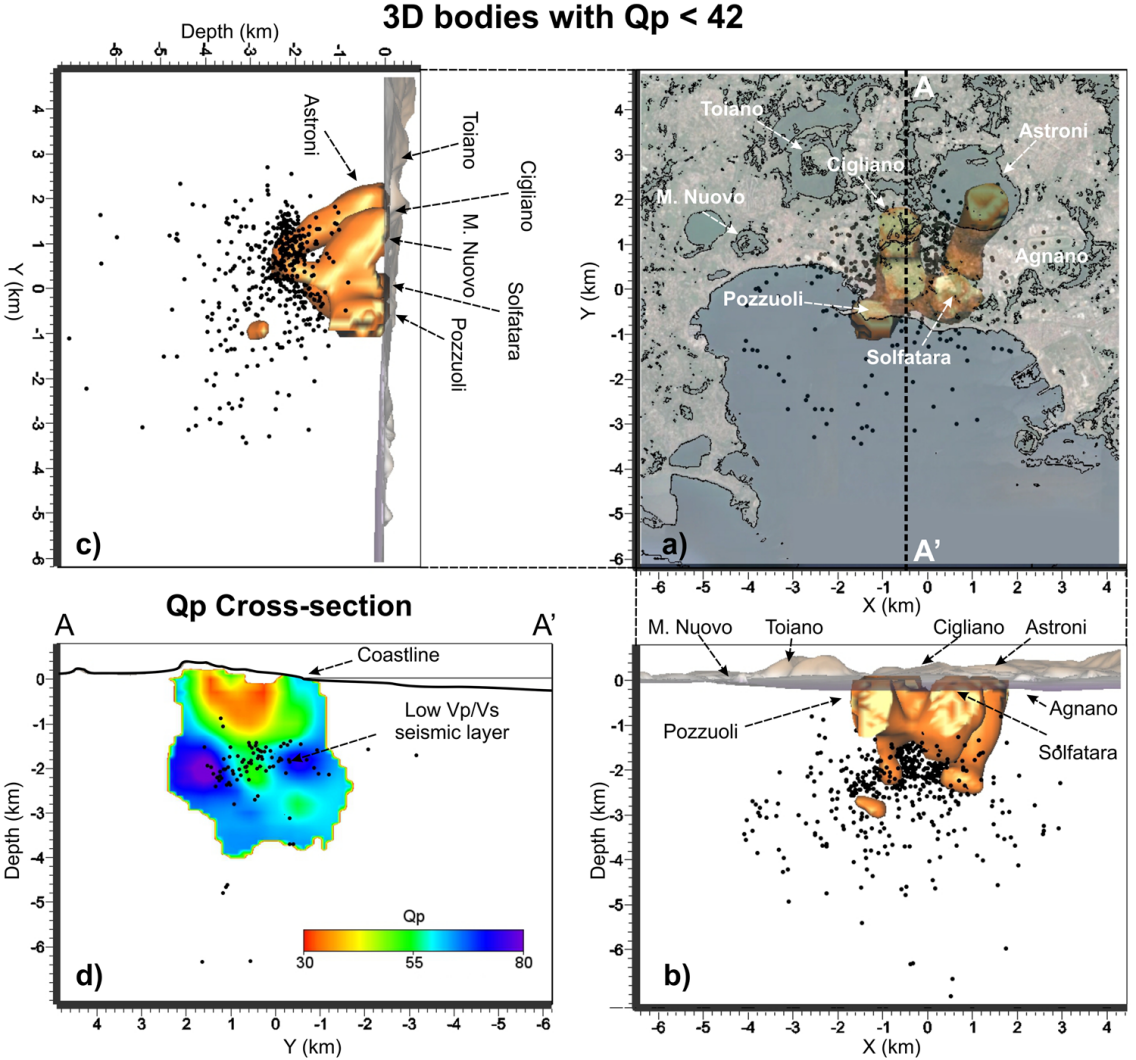
(**a**) Horizontal and (**b**,**c**) vertical projections of the seismicity and of the low Qp bodies with values lower than 42. (**d**) Vertical cross-section of the Qp model along the profile A-A’ that displays the low Qp pattern in the shallow part of the caldera and the part of the seismic layer that probably allows the deep fluids migrating upward. Black dots represent the relocated earthquakes projected onto the vertical slice within ± 0.5 km.

**Figure 6:**
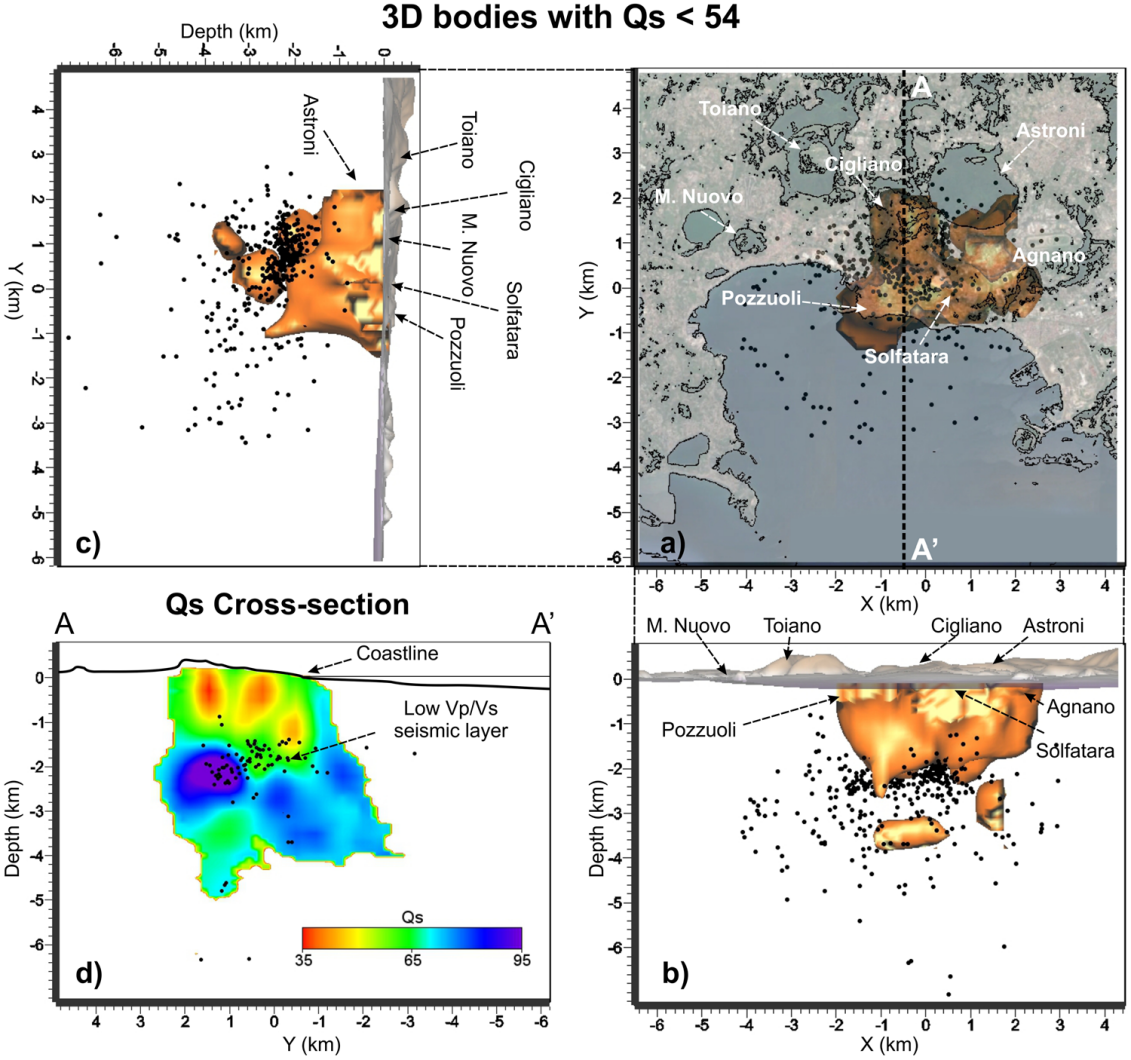
(**a**) Horizontal and (**b**,**c**) vertical projections of the seismicity and of the low Qs bodies with values lower than 54. (**d**) Vertical cross-section of the Qs model along the profile A-A’ that displays the low Qs pattern in the shallow part of the caldera and the part of the seismic layer that probably allows the deep fluids migrating upward. Black dots represent the relocated earthquakes projected onto the vertical slice within ± 0.5 km.

**Figure 7:**
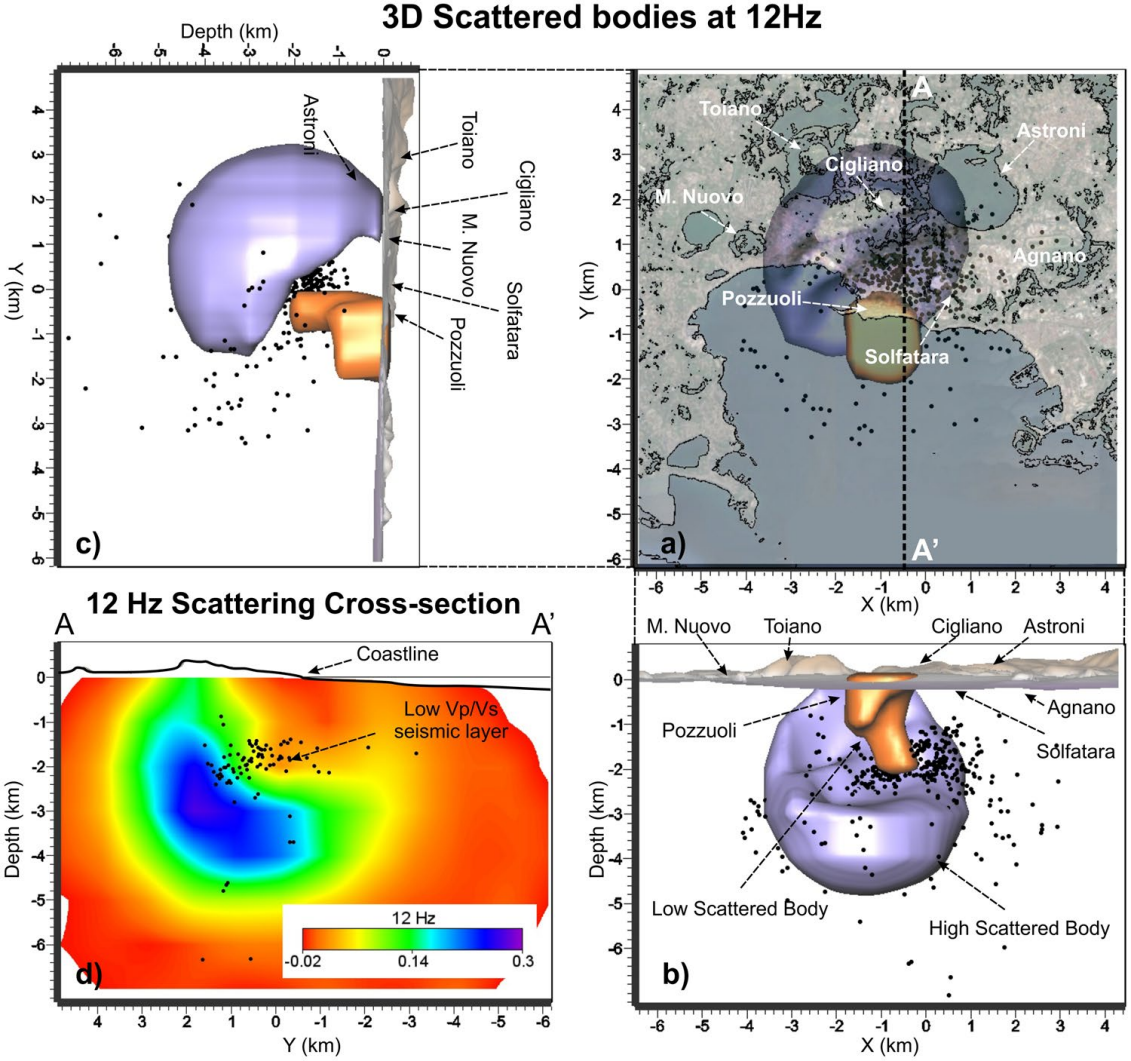
(**a**) Horizontal and (**b**,**c**) vertical projections of the seismicity and of the scattered bodies filtered at dominant frequency of 12 Hz and showing a low scattered body beneath the Pzzuoli region. Higher scattering dominates the country rock. d) Vertical cross-section of the scattering model along the profile A-A’ that displays the pattern of scattered waves. Black dots represent the relocated earthquakes projected onto the vertical slice within ± 0.5 km.

The shallow high Vp/Vs volume below Pozzuoli is also characterized by high P and S attenuation (Qp < 42 Fig. 5; Qs < 54 Fig. 6) and relatively low scattering, down to 2 km depth (Fig. 7). High P attenuations (Qp < 42) and high Vp/Vs values (Vp/Vs > 1.85) are also imaged below the Astroni crater (Figs 2 and 4). Below the Mt Nuovo cone, the Vp/Vs is higher than 1.85 at a shallow depth (0–1 km, Fig. 4a). This feature has never been observed in previous seismic models. The whole inland area is characterized by high scattering at frequencies around 12 Hz, confirming that the medium is generally fractured and extremely heterogeneous (Fig. 2). Our Sc model **(**Fig. 7**)** confirms also that the offshore border of the caldera produces high scattering of the seismic waves, in agreement with^25^, where this pattern was also imaged at higher frequencies (around 18 Hz).

The seismic layer separates the shallow body located beneath Pozzuoli, from a deeper structure located immediately below. This layer is evidenced also at 1.8 km depth in the centre of the Caldera by velocity, attenuation and scattering models. This area, where the seismicity concentrates (Fig. 2 sections B-B’-B” and Fig. 4), includes the region inland of the city of Pozzuoli up to the coastline, extending for more than 4 km^2^. It is about 0.5 km thick, characterized by low Vp/Vs (~1.5, section B-B’-B” of Figs 2 and 4b,c), and marked by relatively low P and S attenuation (Qp and Qs > 70), apart from the central sector where low Qp and Qs extend to greater depths (Figs 2, 5c and 6c).

Below the seismogenic layer, an anomalous body, marked by high Vp (>15%, Fig. 1, 2 and 3), high Vp/Vs (>1.85, Fig. 4), low Qp and Qs values (Figs 2, 5 and 6), and high scattering (Fig. 7), dominates this sector of the caldera. This body has a diameter of about 1 km and extends vertically from 2.3 km to at least 4 km of depth. The upper portion of this anomalous body starts flattening at depths of 2.2–2.7 km assuming a tabular shape 0.5 km thick elongating southward. Sections B-B’-B” of Fig. 2 show this feature for both Vp and Vp/Vs. Attenuation models (Figs 2, 5, and 6) reveal that this body is marked by weak values at least on its upper part.

## Discussion

Thanks to the EST models, we imaged the structure of CF allowing the description of small anomalous bodies located at different depths from the surface down to 4 km.

**Figure 8:**
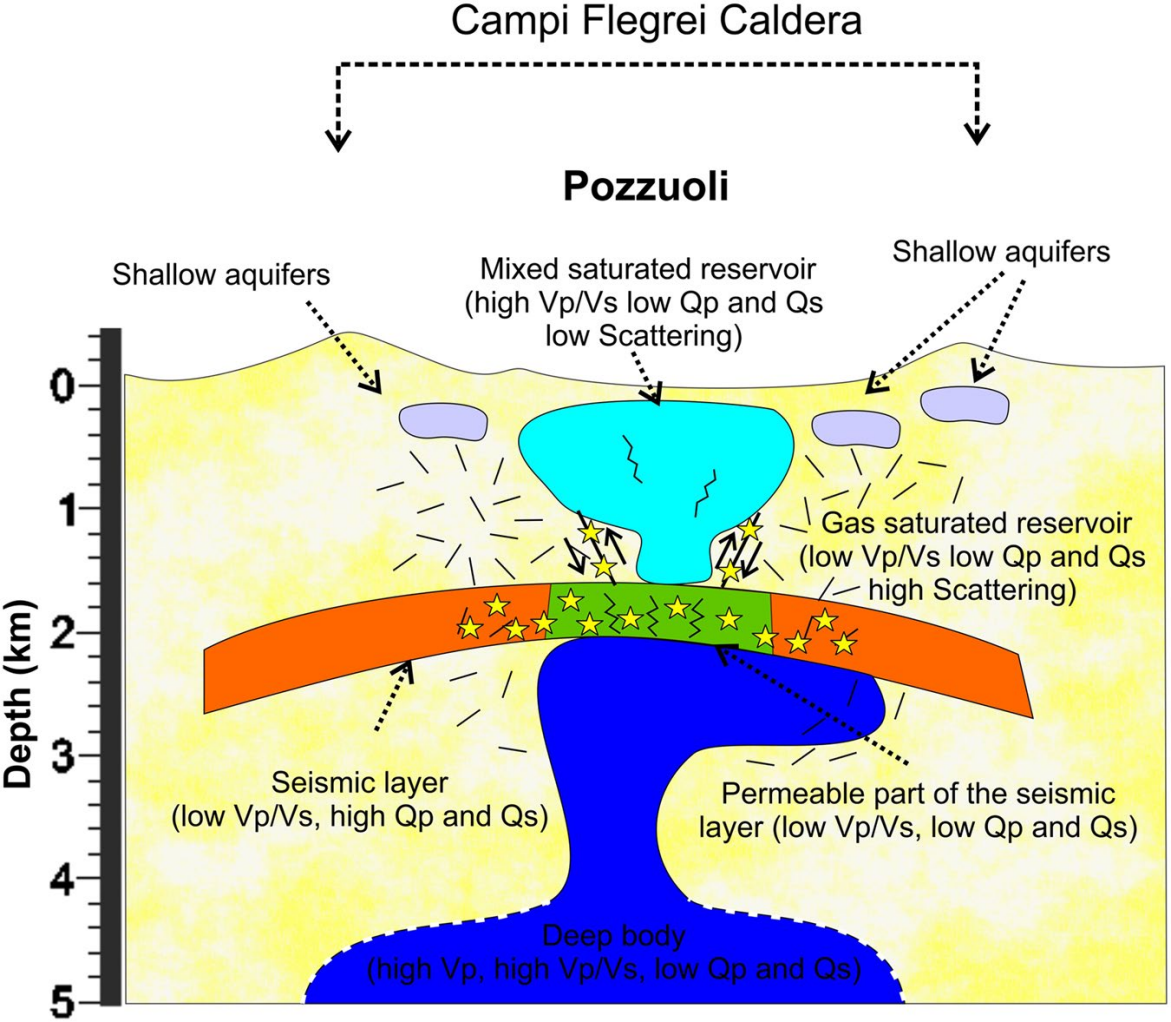
Sketch synthetizing the information retrieved from the P wave Seismic velocities (Vp), Vp/Vs ratio, Quality factor of the P and S waves (Qp and Qs), and Scattering (12 Hz). The seismic layer (marked by low Vp/Vs and high Qp and Qs) separates the shallow part of the caldera from the deeper one. The deep body (marked by high Vp and Vp/Vs and low Qp and Qs) rises from the depth and stops at about 2.3 km where flattens beneath the seismic layer. The most permeable part of the seismic layer allows the migration of magmatic fluids into the shallow part of the caldera where mixtures with the geothermal reservoir beneath the Pozzuoli region (marked by high Vp/Vs and low Qp and Qs and low scattering) by means of shallow active faults driving them toward the surface. The Pozzuoli reservoir is generally bounded by a gas-saturated country rock (marked by low Vp/Vs, Qp and Qs, and high scattering) where also very shallow water saturated reservoirs may exist beneath the main craters of the Caldera.

The sketch of Fig. 8 synthetizes all the features observed in the EST models allowing a joint interpretation of several physical parameters calculated in this study (Vp, Vp/Vs, Qp, Qs, and Sc).

In literature, high Vp values are commonly observed beneath volcanoes^26–28^ and associated with deep material raising trough a magmatic conduit. Other interpretations based on only Vp models infer that those bodies could represent crystalized material^29^. Therefore, the interpretation of the Vp models alone in volcanoes does not solve the paradox. High Vp/Vs values and low Q values are generally associated with regions particularly enriched in water or molten rocks. In CF the geochemical studies performed on gas and water measurements at the Solfatara crater and neighbours wells, indicate that the mixing between magmatic and shallow fluids occurs at no more than 2 km of depth^30^. Following these considerations, the high Vp and Vp/Vs body extending in depth from 2.3 km to 4 km can be associated with a conduit of partially molten material enriched in fluids, excluding, therefore, the possibility to be an old and crystalized plumbing structure. The conduit has a diameter of about 1 km and extends for about 1.5 km in the vertical direction. As observed in our models, and reproduced in the sketch, this body starts flattening at depths of ~2.3–2.4 km assuming a tabular shape. This bending can be ascribed to a change in the physical properties of the overlapping rock that is able to stop its rising toward the surface, forcing it to spread at that depth. This flattened body supports the observation that the maximum deformation is observed inland in the caldera while the actual seismicity is mostly concentrated below the Solfatara area. The position and shape of this body is also in agreement with recent gravity and deformation models^15^. The seismicity occurred in the 1984 concentrated just at the top of the plumbing system, within a thin layer marked by low Vp/Vs. However only shallow permeable faults allow the fluid migration in the Solfatara region (Figs 2 and S1.1 of Supplementary information). These faults are not necessary located above the rising body, as already observed by^10^ and inferred by the geochemical information^5^.

According to recent rock physics analysis performed on core samples collected in the caldera^21^, the bedrock of the CF is characterized by a sort of natural concrete, which increases the strength of the material changing its elastic properties with respect to the surrounding region. Since the rock samples were collected in ancient wells drilled on the caldera rim more than 30 years ago it was difficult to spatially extend the presence of such a layer beneath the whole caldera. The models proposed here allow describing the layer observed in the rock samples extending its presence at least beneath the whole Pozzuoli city at depths of 1.7–2.2 km. This layer is of crucial importance for the stability of the caldera because it separates the shallow aquifer from the deeper structure.

Independent studies e.g^21,31,32^. support the existence of a physical barrier separating the upper and lower sector of the caldera. In our sketch this reinforced layer assumes the key role of stopping the ascending material producing a flattening of the molten rock just below it.

Other authors^20^ imaged a high-to-low attenuation interface suggesting that the deep fluids rising up during the 1984 crisis were trapped by this caprock. In our models we show that the caprock is ~0.5 km thick and mostly marked by high Qp and Qs **(**Figs 5b,d and 6b,d**)**.

Low Qp and Qs, together with a high concentration of the seismicity, characterize the central part of this layer **(**Figs 5b,d and 6b,d and S1.1 of Supplementary information**)**. Presumably, this is the region where the plumbing system pushed upward during the bradyseismic crisis. Because of the changed stress condition, direct faulting occurred^33^ triggering the seismicity observed in the low Vp/Vs layer. This mechanism increased the permeability in some portions of this layer allowing a pathway for the migration of deep fluids toward the shallow part of the caldera, as synthetized in the sketch of Fig. 8. However, fluid migration toward the surface is allowed mostly in regions where permeable faults are reactivated. In our sketch these faults are imaged as structures linking the shallow aquifer and the permeable part of the caprock.

The joint interpretation of the seismic velocities and attenuation models allowed also to discern small anomalous bodies at depths less than 0.8 km and marked by relatively low Vp, high Vp/Vs and low Q (P and S) explainable with the presence of shallow reservoirs mainly water saturated^34–36^. These small bodies are located beneath the largest intra-caldera craters suggesting that the topography plays an important role on the hydrological confluence of the meteoric water feeding these reservoirs.

The shallow high Vp/Vs anomaly (~2.2) observed beneath Pozzuoli is associated with a reservoir where meteoric fluids interact with the magmatic ones^4,22^. As already mentioned, geochemical studies^30^ agree on fixing at about 2 km the depth where the mixing fluids starts. This hypothesis confirms a model requiring a structure located at that depth to regulate the migration of fluids into the shallow reservoir.

Low scattering values support the idea that the Pozzuoli reservoir is mainly water-saturated and/or over-pressured, with open-fractures partially or totally filled by fluids. The fact that the region surrounding the Pozzuoli reservoir is marked by very low Vp/Vs and high-scattered matrix, infers that the bedrock is mostly dry or gas-saturated. The phase state of the bedrock fluids makes the network of fractures highly sensitive to the scattered dispersion phenomena at these depths^9,34^.

According to our results and to the actual geophysical and geochemical knowledge, the mechanism responsible for the fluid mixing can be explained by means of a highly fractured seismic layer. This structure is more permeable in its central part because of the increasing of stress induced by the deep plumbing system that pushed upward during the 80’s. The existence of a physical barrier that changes its permeability as function of the temporal stress variation is at the basis of the cycles of bradyseism. Episodic expansions of the Pozzuoli reservoir due to the fluid and heating exchange between the upper and lower system enhances the local deformation at surface. The clear image we obtained of a magmatic body below this layer can also be considered one of the most important finding of this work. Consequently, the models showed here brightened a new piece of the puzzled structure of the caldera providing a better understanding of the mechanism regulating the unrest periods.

## Methods

The Enhanced Seismic Tomography method combines the application of a well-established approach for seismic data inversion with a post processing called Weighted Average Model (WAM^37–40^), which allows refining the features of the model and reduces the biases that commonly affect an inversion method alone.

### Building of the Vp and Vs seismic models

Seismic velocity models (Vp and Vs) are obtained by applying the Double Difference approach^41^ and the WAM^38,39,42^.

In this case, we developed a three-step procedure to build the EST of the Campi Flegrei consisting in:

#### Refining of the initial event location using a layered Vp and Vp/Vs model

In volcanic regions strong variations of Vp/Vs are expected both laterally and vertically, and the highest resolution is requested to reduce rough errors into the final models. To improve the initial 1D Vp/Vs model we first located the events using the initial 1D Vp model used in literature for the CF^9,23,24^ and a constant Vp/Vs. Then, the hypocentres were separated in sets as function of the depth. We calculated an average Vp/Vs value for each set of events using the Wadati diagrams^43^. We then obtained an optimized distribution of the Vp/Vs in depth using (a) the Wadati values obtained for each set of events, and (b) the information coming from the existing models e.g^9,18,23,24^. (see Fig. S1.2 in Supplementary information). The optimized 1D Vp/Vs model is finally used together with the 1D Vp model to calculate again the hypocentre parameters obtaining new event locations. The improved locations, the 1D Vp and the new 1D Vp/Vs model, are used as input for our tomography inversions.

#### Double Difference (DD) tomography

In this step we applied the DD tomographic method developed by^41^. The algorithm determines 3D velocity models jointly for P and S waves with absolute and relative event locations. This approach has the advantage of including relative arrival times calculated between pairs of events with their quality values along with absolute arrival times, thereby not discarding valuable information by using only adjusted picks. At the same time, this approach avoids simplified assumptions about ray path geometries or path anomalies producing absolute, and not just relative, locations^41^. In this work we used the data recorded by 21 three-component digital stations, installed by the University of Wisconsin between January and April 1984 in the CF area during a bradyseismic crisis^13^. The data were recorded with different sampling rates, which were sometimes changed during the field surveys. To provide a uniform dataset, all the records were resampled at 100 samples per second (sps), the lowest sampling rate. For the seismic velocity models we selected 591 well-recorded events with at least 5 P-waves and 4 S-wave pickings for a total of 3,351 P and 2,508 S phases. The dataset was complemented with 34,593 P and 21,047 S differential times calculated between pairs of events having inter-event distances less than 1 km. The distance between strongly linked events resulted on average 0.7 km. This approach results in a dramatic increase of data used to obtain the final models with respect to the classic travel time tomography inversion methods.

In this work we imposed 20 iterations for the joint inversion of velocity and event location. We adopted a weighting scheme that provides high weights to the absolute data in the first 5 iterations and high weights to the differential ones in the following 5. The scheme is repeated twice. Finally we performed 5 additional iterations imposing the event re-location only to further refine the hypocenters into the 3D models.

#### Weighted Average Model (WAM)

In this last step we refined the final Vp and Vs models applying the WAM method^37,38,40,42^. WAM is a post-processing technique that may be used with any tomographic inversion method to overcome some common limitations of the models obtained with the standard tomographic codes. Although DD models are considered better resolved than those obtained with the standard techniques, they are not immune to the traditional problems affecting the tomographic inversions. Hence, the initial parameters that are imposed to obtain the 3-D velocity models (e.g. initial velocity model, model parameterization, data selections, etc.) may strongly affect the results.

The WAM method is based on different sampling of the models, compatibly with the data sets, performed by imposing different input parameters. The results are then merged in a new and more reliable model using weighting functions based on the ray density Derivative Weight Sum, (DWS^44^), of each model. In our case, we resampled the space of the velocity models 13 times; each time it was either rotated by a small angle (eight times) or translated by 1/3 of a grid distance to the sides and in depth (five times). Moreover, the translation of the grid in depth produced a perturbation of the initial 1D Vp and Vs models so that the perturbations of these parameters are also considered in the procedure (see Fig. S1.3 in Supplementary Information).

The 13 Vp and Vs models were re-sampled in a common Cartesian grid (WAM grid^37^). The origin of the WAM grid is located at the point of coordinates 40.828 N and 14.136 E and 0.5 km a.s.l. Its size is 11 × 11.2 × 8.2 km in X, Y and Z directions, respectively and horizontal and vertical spacing of 0.15 km. Corresponding DWS values were also interpolated in the nodes of the same grid and used for the weighting of the models. In this work, the WAM velocity distributions are based only on seismic velocities calculated on nodes with DWS > 50, as used in other small-scale studies (e.g.^45–47^) to ensure a good resolution of the velocity pattern. Furthermore, the Vp/Vs model has been calculated by dividing directly the obtained Vp and Vs values.

We performed several synthetic tests to show that the method and data are able to finely recover structures of 0.5 × 0.5 × 0.5 km^3^, vertically elongated bodies with section of 1 × 1 km^2^ and flat bodies 0.5 km thick. In these tests we also evaluated the grade of smearing that could affect the velocity pattern and the amount of the data noise needed to limit a fair interpretation of the recovered structure (see figures from Figs S1.4 to S1.6 of the Supplementary Information).

### Building of the Attenuation models (Qp and Qs)

Attenuation models are obtained by applying a similar procedure. In this case we skipped the step N 1, and in step N 2 we used the code Simulps13q^48^, which incorporates a t* inversion routine to determine the 3D distribution of damping values^49^.

The non-dimensional seismic quality factor, Q, that we estimated starting from a frequency independent t* parameter, is calculated on displacement waveforms where instrumental and site responses have previously been removed^50,51^. The amplitude spectra for both P and S waves are obtained applying the fast Fourier transform. We considered a window of 1.5 s around the P wave picking and 3 s around the S wave one to evaluate the amplitude spectra. From our tests and from bibliography^52^, these windows are considered sufficiently long to smooth the radiation pattern effects and to guarantee a stable estimation of the t* parameter.

Once deconvolved by site and station contributions, the theoretical displacement spectra can be written as a function of frequency *f* as equation (1):

1 $$H(f)=\frac{{{\rm{\Omega }}}_{0}}{{[1+{(\frac{f}{{f}_{c}})}^{\gamma }]}^{1/2}}\exp (-\pi {t}^{\ast }f)$$

where ${{\rm{\Omega }}}_{0}$ is the low frequency spectral asymptote, *f*_*c*_ the corner frequency and *γ* the source spectral fall-off ^53^. Here we assumed a Brune source, $\gamma$ = 2^54^ and a frequency independent t*. By fitting the corrected displacement spectrum with *H(f)* in the frequency band 2–20 Hz we estimated ${{\rm{\Omega }}}_{0}$, *f*_*c*_ and t* using a grid search method. The *f*_*c*_ value is constrained around 0.37*β*(0.4375*M_0_*/Δσ)* where *β* is the S-wave velocity, *M*_*0*_ the seismic moment and *Δσ* the stress drop^54,55^. *β* and *Δσ* are assumed equal to 2 km/s and 5 bar, respectively^33^. Since the dataset used to carry out the model is similar to that of ^52^, we refer the reader to it for further details.

In order to obtain the highest quality of Qp and Qs distributions we calculated 1334 t* for P and 1322 t* for S waves from 293 events located with the WAM velocity models.

Similarly to the seismic velocity models procedure, we applied the step N 3 adopting the WAM approach. We then calculated 13 models with the same inversion grids as for the velocity models and merged them using weighting functions based on the DWS parameter (see Supplementary Information).

### Building of the Scattering model

Similarly to the attenuation models we first used the procedure described by^25,56^ and based on the method proposed by^57^. This scattering locator method considers the experimental evidence that the coda energy density can be modelled on a first order with a single isotropic scattering approach. By assuming a medium filled with a random and uniform distribution of scatters, the variation in the exponentially decaying envelope is assumed to be induced by the energy singularly scattered by the heterogeneities/scatters located in the medium. The lapse times of the bumps in the coda envelope are related to the scatter positions, as described in^25^. For each earthquake recorded at a station the mean envelope is estimated as the best fitting exponentially decaying curve. Each deviation from the fitting envelope is assumed as possible scattered energy due to a scattered located in each position for which the travel time is equal to the observed time lapse. Further details on the procedure used for retrieving the data are described in^25^. In this case, the medium is gridded in boxes of 1 × 1 × 1 km^3^ and the envelope variations are estimated in windows of 0.5 s starting from 2 times the S waves arrival times. To each box a high number of possible anomalies are associated, the mean of them is the tomographic value. The higher the associated value, the best is the resolution of the method in the area. In this case, to build the WAM model, we estimated 8 models with the grid shifted both horizontally and vertically of 1/3 of the box side.

## Electronic supplementary material

Supplementary information

## Data Availability

The data that support the findings of this study are available on request from the INGV.
