# Supplementary material for: Anatomy of the Campi Flegrei caldera using Enhanced Seismic Tomography Models
Source: Sci Rep. 2018 Nov 2;8:16254. doi: 10.1038/s41598-018-34456-x (PMC6214947; doi:10.1038/s41598-018-34456-x)
Supplement: Supplementary file 1 — Supplementary information [file 41598_2018_34456_MOESM1_ESM.pdf]

SUPPLEMENTARY INFORMATION

Anatomy of the Campi Flegrei caldera using Enhanced Seismic Tomography Models

*Marco Calò[1] and Anna Tramelli[2]

1) Universidad Nacional Autónoma de México, Instituto de Geofísica,

*Corresponding author: calo@igeofisica.unam.mx

2) Istituto Nazionale di Geofisica e Vulcanologia sez. Napoli - Osservatorio Vesuviano

	Page
1) Maps with the final location of the seismicity	2
2) Set up of the 1D models Vp , Vs and Vp/Vs	3
3) Set up of the WAM models from the inversion of 13 perturbed grids.	5
4) Assessment of the Vp and Vs models.	5
5) Assessment of the model Qp, Qs and Scattering model.	11
6) References.	13

1) Maps with the final location of the seismicity.

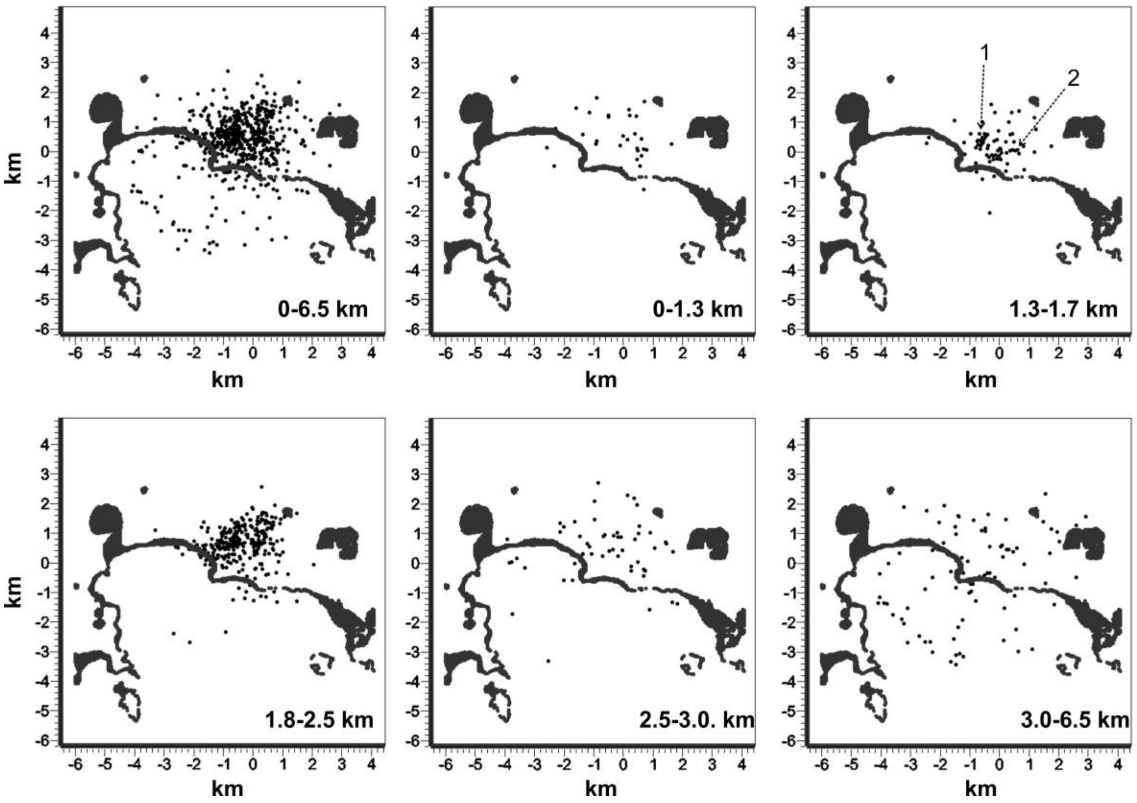

Figure S1.1. Maps of the whole seismicity located with the final 3D seismic models and slices at different depth ranges.

2) Set up of the 1D models Vp , Vs and Vp/Vs.

Figures **S1.2a** and **S1.2b** show the initial 1D models of Vp, Vs (black and red in pannel **a**) and Vp/Vs (black in **b**) used for the preliminary events location and for the Double Difference tomography inversions. The initial Vp model is the same as used by **[1, 2, 3, 4, 5]** for their tomographic studies. The Vp/Vs model has been obtained from the results of the previous tomographic models **[1, 2, 3, 4, 5]** and updated calculating the Wadati diagrams of the events falling into the depth range where a significant change of the Vp/Vs ratio has been observed. Finally, the events are re-located with the Vp and the new Vs model using a modified version of the location code Hypo71pc **[6].** The average Root Mean Square (RMS) obtained for the new locations resulted of 0.13s. **Figure S1.2c** shows the horizontal and vertical projections of the P-wave ray path coverage using the new preliminary locations. The average RMS of the relocated events on the final 3D WAM models is of 0.048s, resulting in an improvement of the hypocenter pattern that is twice with respect to that obtained with the previous models that reached a minimum value of 0.08s **[2, 3, 4]**.

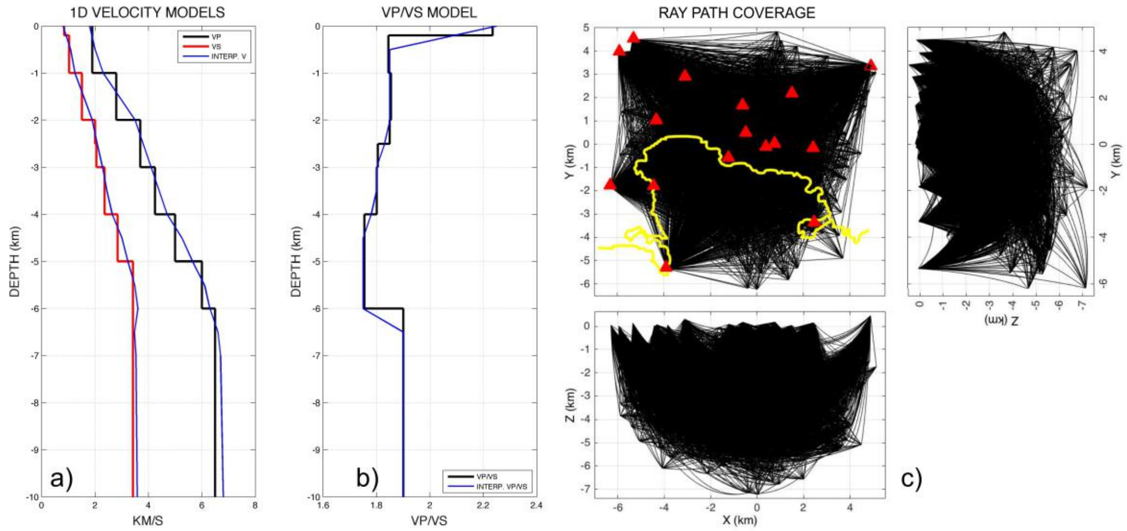

Figure S1.2. a) Layered Vp (black) and Vs (red) models used for the preliminary location and interpolated models (blue) used as starting velocities for the double difference tomography inversions. b) Layered Vp/Vs distribution in depth (black) and corresponding interpolated model (blue). c) Horizontal and vertical projection of the P-wave ray path coverage of the studied area.

3) Set up of the WAM models from the inversion of 13 perturbed grids.

Figure S1.3 shows the horizontal and vertical density sampling of the investigated volume obtained merging the 13 grids used for the 13 Double Difference tomographic inversions that have been generated to build the WAM. It is worth noting that the shift of the grid in depth produces small perturbations of the initial Vp and Vs models increasing the variability of the space of the models sampled during the inversions. The figure shows the difference in the density of points sampled with the WAM rather than using a grid alone.

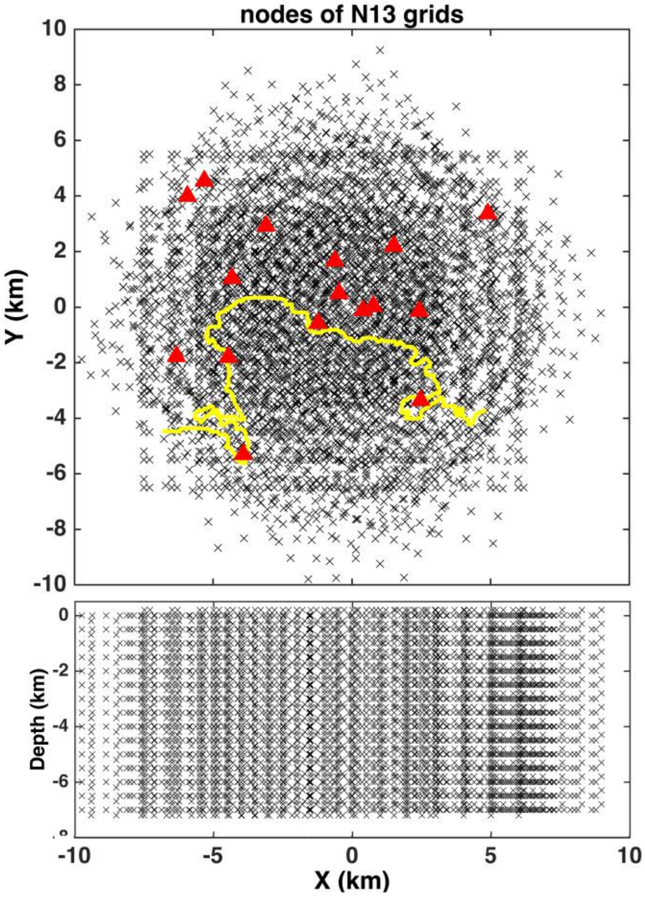

Figure S1.3. Horizontal and vertical projection of the 13 inversion grid points used to build the final model with the WAM method. Red triangles are the position of the stations used and the coastline is reported in yellow.

4) Assessment of the Vp and Vs models.

To assess the reliability of our results and to show the resolution power of the data and method, we constructed a checkerboard model characterized by alternating positive and negative velocity changes of ±10 percent with respect to the initial 1-D velocity model. Each patch has size of 0.5 km along the three dimensions. The model was then used to calculate synthetic travel times using the same configuration of earthquakes and stations as in the real inversion. Travel time errors were simulated by adding Gaussian distributed noise with a standard deviation of 0.04s and 0.06s to the P and S arrival times, respectively, corresponding to a 2σ of the largest expected picking error for this dataset **[3]**.

The 1-D initial model described above was then used as the starting velocity structure for the inversion of the perturbed synthetic database applying the Double Difference and the post-processing (WAM). **Figures S1.4a** and **S1.4b** show the results of checkerboard test for Vp and Vs models at depth of 0.75 km, 1.75 km and 2.75 km, and four cross-sections (two E-W and two N-S).

The best resolved areas are located in the center of the study region, with a depth ranging between 0 and 4.5 km, in correspondence with the largest ray path lengths and densely criss-crossed grid cells. In the cross-sections only the part of the volume with Derivative Weight Sum (DWS**, [7]**) larger than 50 is shown.

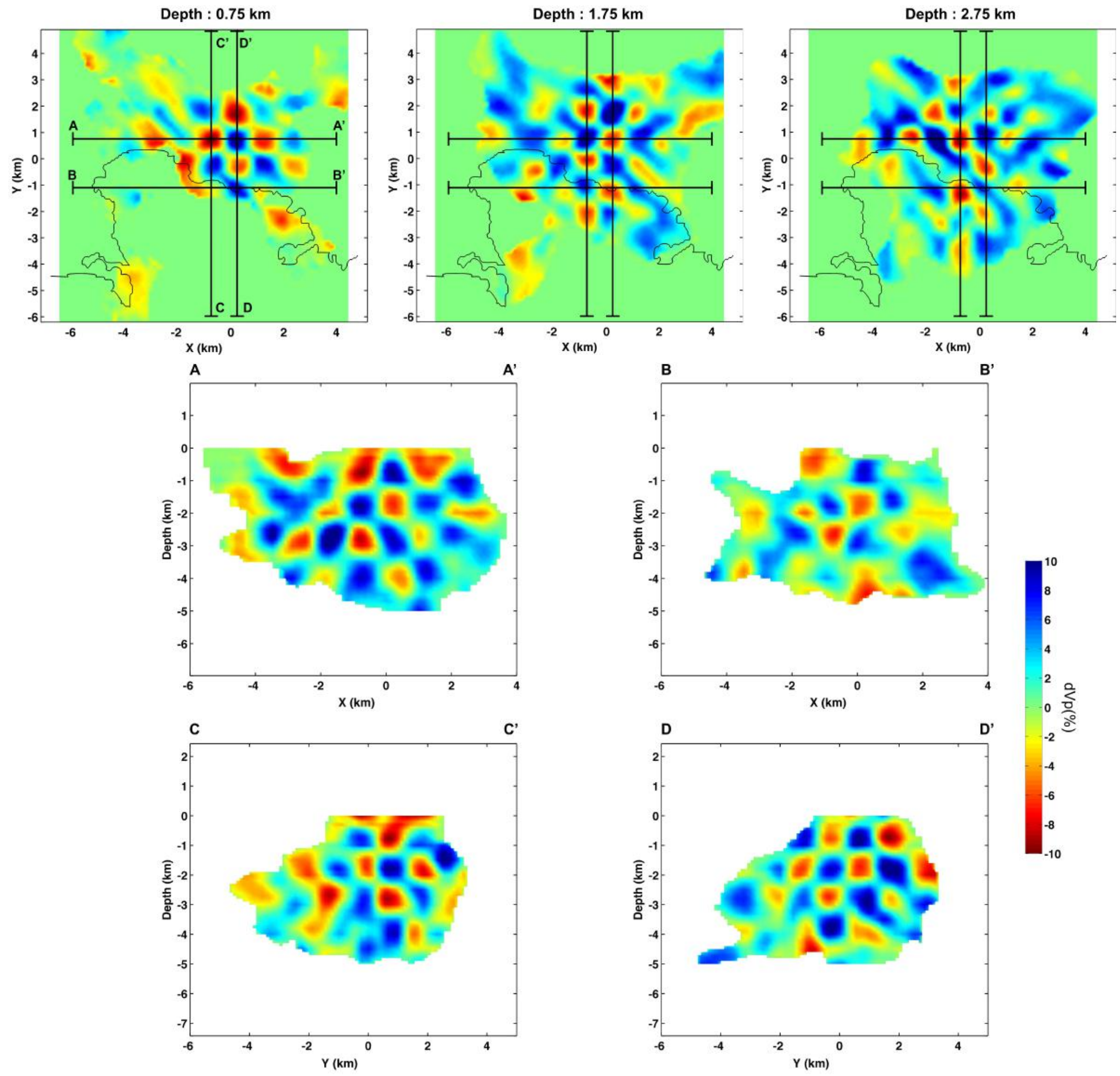

Figure S1. 4a. Horizontal and vertical sections of the P wave checkerboard test. The horizontal projections are shown for depths of 0.75 km, 1.75 km and 2.75 km. The intersection between the vertical projections and the horizontal planes are indicated in the top panels.

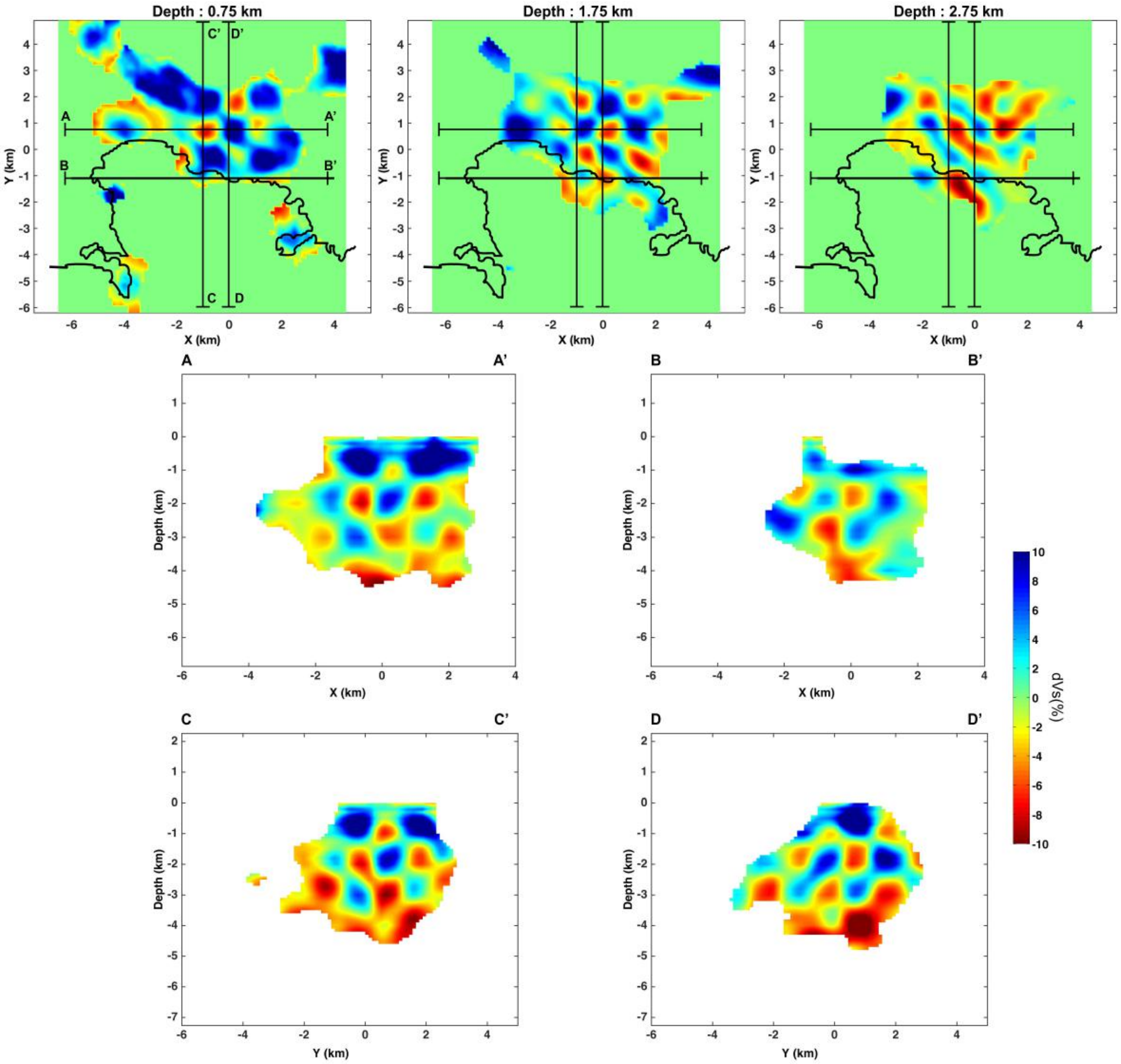

Figure S1.4b. The same as Fig. S1.4a but for S-wave checkerboard test.

5) Spike tests

To further assess the reliability of the structures observed in the models obtained using the real data, we performed two discrete spike tests or sparse checkerboard tests **[8]** that involve a sparse distribution of spikes (**figure S1.5a** and **S1.5b**). These tests were performed to evaluate 1) the magnitude of smearing for near features **[8]**, and 2) the level of data noise needed to degrade the tomographic images with the consequence of limiting the interpretation of the models.

Figure S1.5a shows the spike test performed with levels of noise of 0.04s and 0.06s for the P and S arrival times, respectively. The method allows to finely recover the spikes and, as evident, the smearing effects are very reduced. These levels of noise are 2σ of the largest expected picking error for this dataset **[3]**.

Figure S1.5b shows that only with travel time errors about 8 times greater than the ones expected for the CF (4 if we consider a 2σ of the errors), the models start to be degraded to levels where the reconstruction of the spikes is poor, especially at depths grater than 2.5 km. However so big errors on the pickings (0.08s for P and 0.14s) would imply a

miscomputation in the travel times of almost 10% of their values and an initial error of the event locations of more than 2 km. These conditions are unrealistic for the CF database. Therefore, this test demonstrates that with the used data it is difficult to introduce biases into the velocity models large enough to lead to a misinterpretation of the recovered structure.

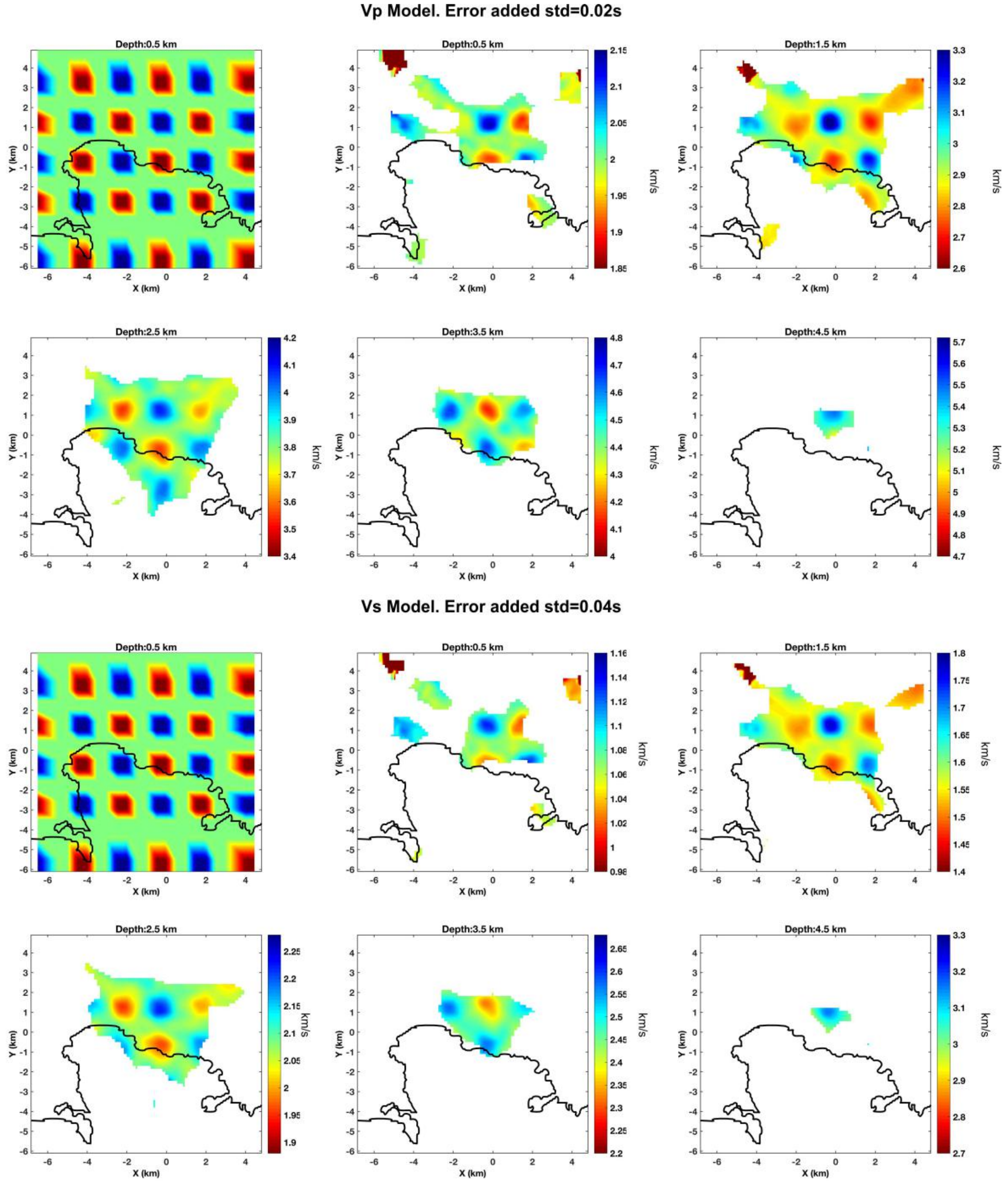

Figure S1.5a. Horizontal sections of the P- and S-wave discrete spike tests using a realistic level of noise of the data. Top left models are the pattern of the true model. Note that the layer at 3.5 km depth has a pattern inverted with respect to the other ones. The true model was built with this feature to further increase the complexity of the model.

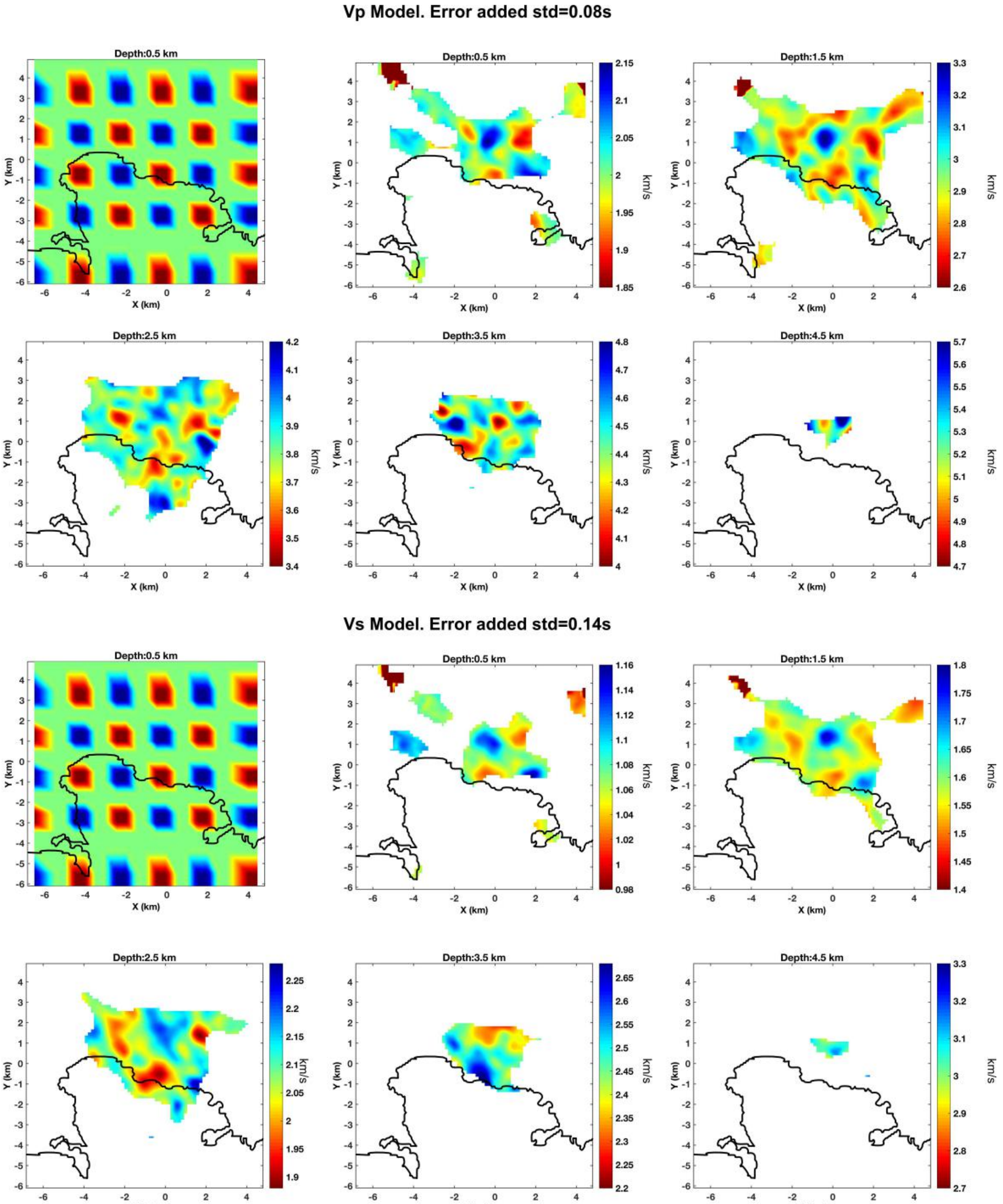

Figure S1.5b. Horizontal sections of the P- and S-wave discrete spike test using level of noise of the data four times than the expected 2 errors. Top left models are the pattern of the true model. Note that the layer at 3.5 km depth has a pattern inverted with respect to the other ones. The true model was built with this feature to further increase the complexity of the model

6) Restoration–Resolution test

Finally, to assess the reliability of the deep structure observed, we built a synthetic model characterized by a P- and S-wave velocity anomaly 25% higher with respect to the initial 1-D velocity model. The anomalous body has the shape of a rectangular prism with horizontal section of 1x1 km^2 at 5 km depth flatting at 3 km depth into a shallow body

0.5 km thick elongating about 2 km S-W and 0.7 km large (**figures S1.6a, b**, and **c**). This model can be considered as an approximation of the velocity anomalous structure that has been observed in the inversions done with the real data, allowing us to perform a Restoration–Resolution test **[9]**. With the same configuration of earthquakes and stations as in the real data inversion, we calculated synthetic travel times, adding picking errors of…., and running an inversion procedure as for the real data. Results show that the data and method used are able to fairly recover such a complex structure down to 4.5 km depth for both Vp (**figures S1.6d, e**, and **f**) and Vs models (**figures S1.6g, h,** and **i**).

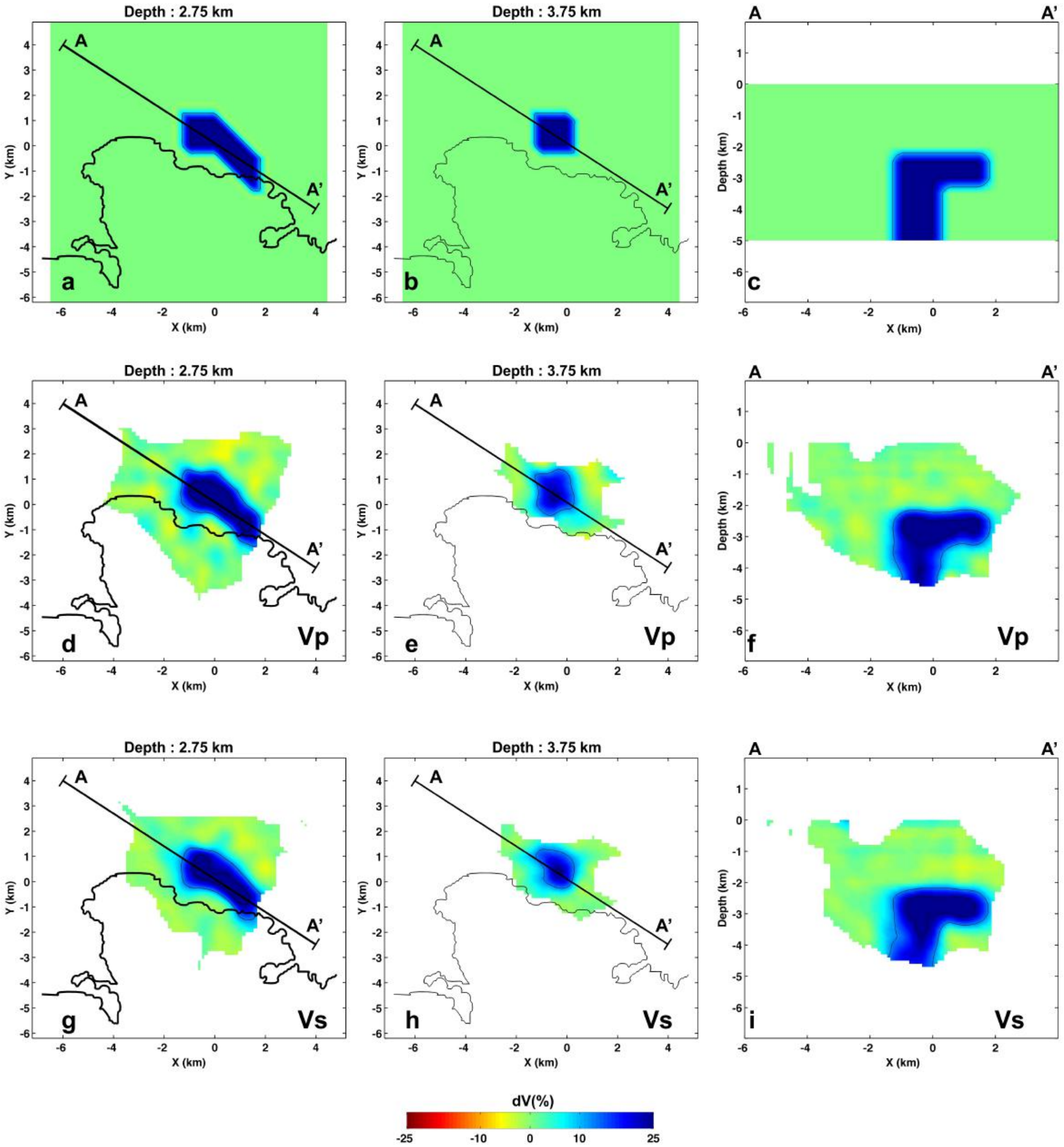

Figure S1.6. a,b,c) Model used to calcualte the synthetic P and S travel times, d,e,f) results for the P wave tomography, g,h,i) results for the S wave tomography.

Following all the tests and considerations done, we can affirm that the velocity models presented here are able to fairly reconstruct features with linear dimension of 0.5 km in the CF for depths ranging from 0 km to about 4.5 km. The possibility that these models contain artifacts that could be misinterpreted is considered low in the best resolved regions.

7) Assessment of the model Qp, Qs and Scattering model.

The reliability of the Qp and Qs structure was assessed using the DWS parameter **[8]**, which is related to the amount of ray paths passing near the nodes of the inversion grid. Several authors relate the DWS to the resolution and the reliability of the structures recovered for both velocity and attenuation models **[10, 11, 12, 13, 14, 15]**. The lower threshold of this parameter is used for delimiting the best-resolved region and depends on several factors such as the size of the grid, the amount of stations and events, their spatial distribution, etc. In literature DWS goes from small values (e.g. DWS=10, **[14, 16, 17]** to DWS=100 and more **[11, 18, 19]**. In this work we adopted a threshold of 100 for delimiting the best-resolved volume (**figures S1.7a** and **S1.7b**), which is twice that generally used in other attenuation studies of similar size and amount of data (e.g. **[10])**. Furthermore we interpret only the part of the models falling into the regions where the DWS is greater than 500 to ensure the best reliability of our interpretation.

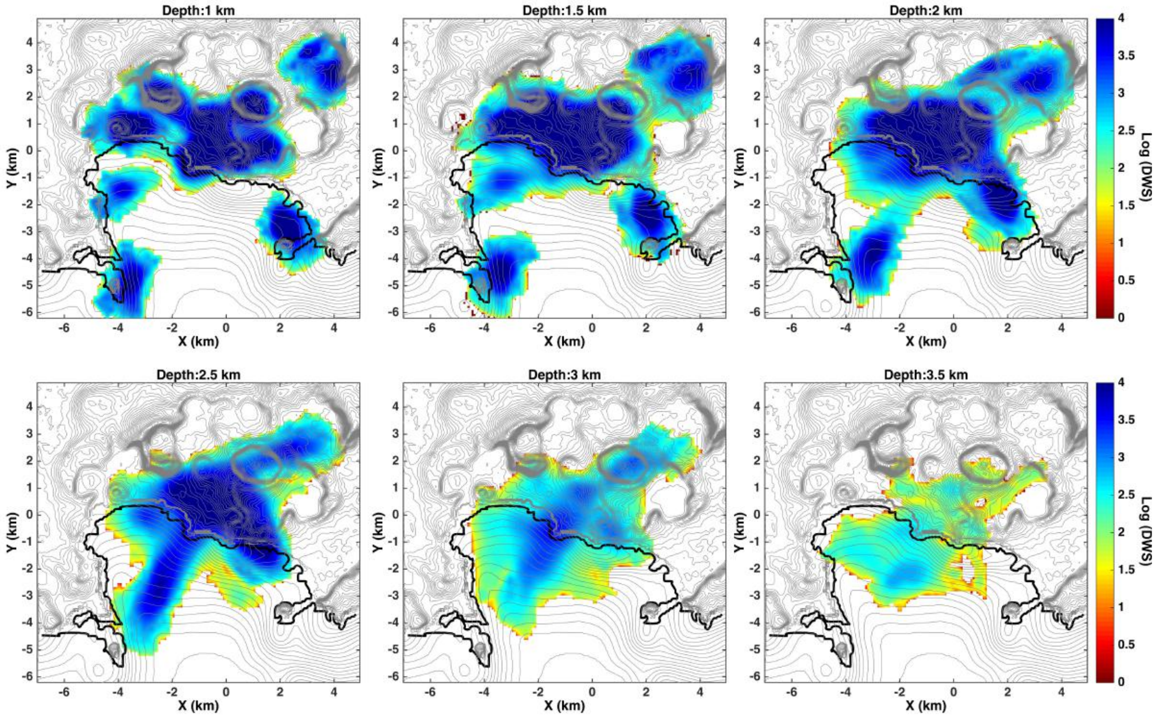

Figure S1.7a. Derivative Weight Sum (DWS) for the Qp data at different depths.

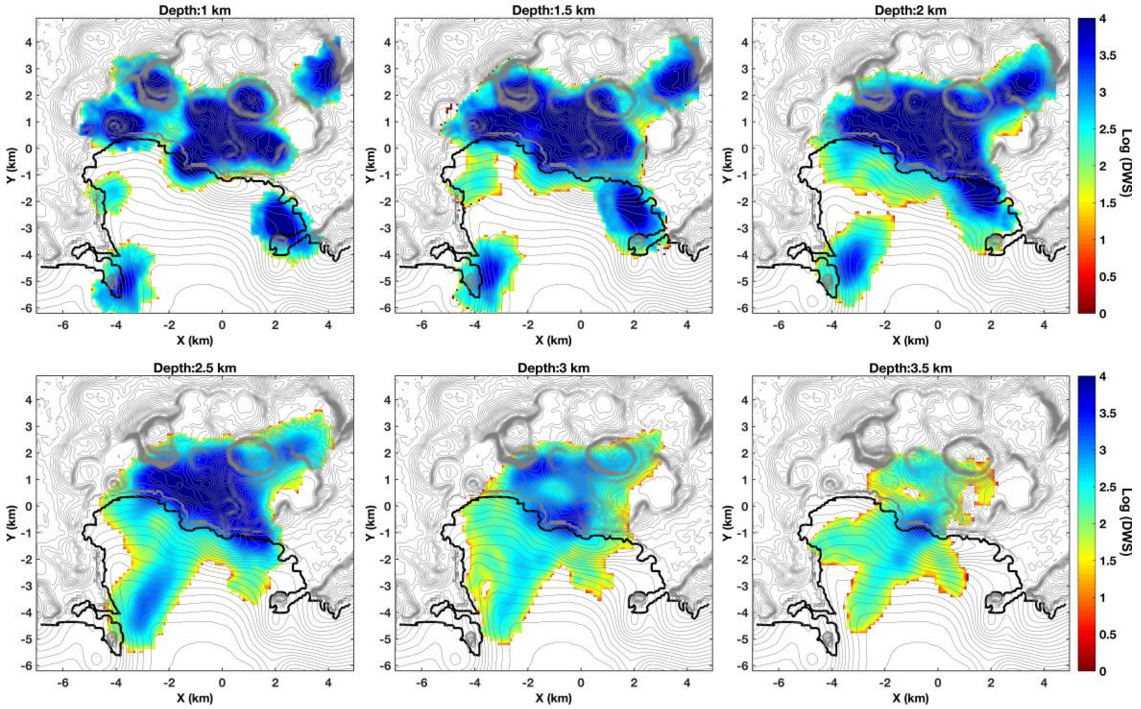

Figure S1.7b. Derivative Weight Sum (DWS) for the Qs data at different depths.

The reliability of the scattering model has been assessed using the data coverage for each grid cell, i.e. the number of possible scatters associated with each volume during the analysis. **Figure S1.7c** shows the number of scatters obtained for the envelopes filtered at the dominant frequencies around 12 Hz calculated at different depths delimiting the region of influence of the asperities.

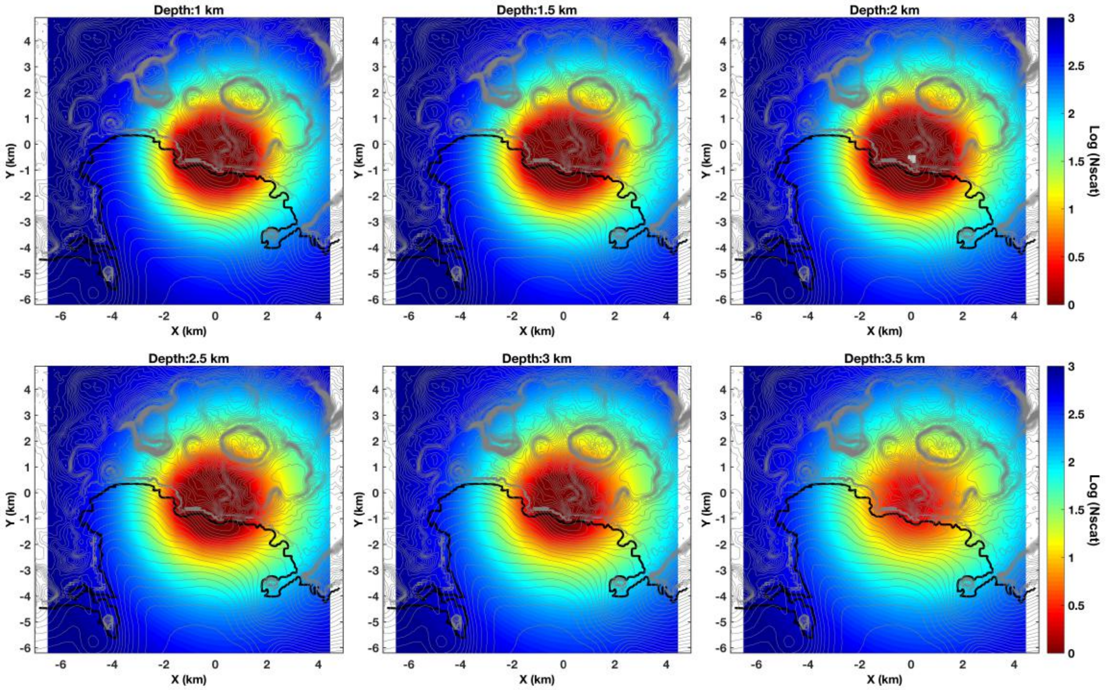

Figure S1.7c. Number of scatters calculated at different depths.

8) References.

1. Zollo, A. *et al*. Evidence for the buried rim of Campi Flegrei caldera from 3-d active seismic imaging. *Geophys. Res. Lett*. **30**, 19; 10.1029/2003GL018173 (2003)

2. Vanorio, T., Virieux, J., Capuano, P. & Russo, G. Threedimensional seismic tomography from P wave and S wave microearthquake travel times and rock physics characterization of the Campi Flegrei Caldera, *J. Geophys. Res*., **110**, 1-14 (2005)

3. Chiarabba, C. & Moretti, M. An insight into the unrest phenomena at the Campi Flegrei caldera from Vp and Vp/Vs tomography. *Terra Nova* **18 (6)**, 373–379 (2006)

4. Battaglia, J., Zollo, A., Virieux, J. & Dello Iacono, D. Merging active and passive data sets in traveltime tomography: The case study of Campi Flegrei caldera (southern Italy), *Geophys. Prospect.* **56**, 555–573 (2008)

5. Priolo, E. *et al.* The Campi Flegrei Blind Test: Evaluating the Imaging Capability of Local Earthquake Tomography in a Volcanic Area. *Int. J. of Geophysics* **505286**,1-37;10.1155/2012/505286 (2012)

6. Lee, W.H. & Valdes C.M. HYPO71PC; a personal computer version of the HYPO71 earthquake location program. *Open-File Report* **85**-749 (1985)

7. Toomey, D.R. & Foulger, G.R. Tomography inversion of local earthquake data from the Hengill: Grensdalur central volcano complex, Iceland, *J. Geophys. Res.* **94(B12)**, 17497–17510 (1989)

8. Rawlinson, N. & Spakman W. On the use of sensitivity tests in seismic tomography, *Geophys. J. Int.* **20(2)**, 1221–1243, https://doi.org/10.1093/gji/ggw084 (2016)

9. Zhao, D., Hasegawa, A., Kanamori, H. Tomographic imaging of P and S wave velocity structure beneath north-eastern Japan. *J. Geophys. Res.* **97**, 19909–19928; 10.1029/92JB00603 (1992)

10. Amoroso, O. *et al*. From velocity and attenuation tomography to rock physical modeling: Inferences on fluid-driven earthquake processes at the Irpinia fault system in southern Italy, *Geophys. Res. Lett.* **44**, 6752–6760; 10.1002/2016GL072346 (2017)

11. Bisrat, S.T., DeShon, H. R., Pesicek, J., & Thurber, C. High-resolution 3-D P wave attenuation structure of the New Madrid Seismic Zone using local earthquake tomography, *J. Geophys. Res*. **119**, 409–424; 10.1002/2013JB010555 (2014)

12. Calò, M., Parisi, L. & Luzio D. Lithospheric P and S wave velocity models of the Sicilian area using WAM tomography: procedure and assessments, *Geophys. J. Int.* **195(1)**, 625–649; 10.1093/gji/ggt252 (2013)

13. Gritto, R., Yoo, S.H., Jarpe, S.P. Three-Dimensional Seismic Tomography At The Geysers Geothermal Field, Ca, Usa, *Proceedings, Thirty-Eighth Workshop on Geothermal Reservoir Engineering Stanford University*, SGP-TR-198 (2013)

14. Dorbath, C., Gerbault, M., G., Carlier & Guiraud, M. Double seismic zone of the Nazca Plate in northern Chile: High-resolution velocity structure, petrological implications, and thermomechanical modeling, *Geochem. Geophys. Geosyst.* **9**, Q07006; 10.1029/2008GC002020 (2008)

15. Zhang, H. & Thurber, C. H. Estimating the model resolution matrix for large seismic tomography problems based on Lanczos bidiagonalization with partial reorthogonalization. *Geophys. J. Int.* **170**, 337–345; 10.1111/j.1365-246X.2007.03418.x (2007)

16. Zhang, H. *et al*. High-resolution subducting-slab structure beneath Northern Honshu, Japan, revealed by double-difference tomography, *Geology*, **32(4)**, 361-364 (2004)

17. Zhang, H. & Thurber, C.H. Double-difference tomography: the method and its application to the Hayward fault, California, *Bull. Seism. Soc. Am.* **93**, 1175–1189 (2003)

18. Calò, M., Dorbath, C., Luzio, D., Rotolo, S.G. & D'Anna, G., Seismic velocity structures of southern Italy from tomographic imaging of the Ionian slab and petrological inferences, *Geophys. J. Int.* **191**, 751–764 (2012)

19. Eberhart-Phillips D. Northern California Seismic Attenuation: 3D QP and QS Models. *Bull. Seism. Soc. Am*. **106(6),** 2558–2573; https://doi.org/10.1785/0120160060
